# *catena*-Poly[[[tetra­aqua­cobalt(II)]-μ-4,4′-bipyridine-κ^2^ *N*:*N*′] 2-[4-(2-carboxyl­ato­eth­yl)phen­oxy]acetate]

**DOI:** 10.1107/S1600536809021552

**Published:** 2009-06-13

**Authors:** Xi-Fang Wang, Chong-Bo Liu, De-He Huang, Zhi-Qiang Xiong

**Affiliations:** a School of Environment and Chemical Engineering, Nanchang Hangkong University, Nanchang 330063, People’s Republic of China; b Instrumental Analysis Center, Nanchang Hangkong University, Nanchang 330063, People’s Republic of China

## Abstract

In the title complex, {[Co(C_10_H_8_N_2_)(H_2_O)_4_](C_11_H_10_O_5_)}_*n*_, the unique Co^II^ ion lies on an inversion center and is coordinated by two N atoms from two 4,4′-bipyridine ligands and four O atoms from four water mol­ecules in a slightly distorted octa­hedral coordination geometry. The 4,4′-bipyridine ligands bridge Co^II^ ions into a one-dimensional chain structure. In the crystal structure, inter­molecular O—H⋯O hydrogen bonds link cations and anions into a three-dimensional network. The dianions are completely disordered about an inversion center.

## Related literature

For background to assembly of high-dimensional supra­molecular coordination polymers, see: Ye *et al.* (2005 ▶). For 3-(4-hydroxy­phen­yl)propanoic acid as a potential multidentate ligand and a good donor and acceptor of hydrogen bonds, see: Tan *et al.* (2007 ▶). 4,4′-Bipyridine is widely used as a spacer in the construction of supra­molecular architectures, see: Tao *et al.* (2000 ▶); Cussen *et al.* (2002 ▶). For the analogous one-dimensional structure with a 3-carboxyl­atophenoxy­acetate dianion, see: Zhao *et al.* (2005 ▶).
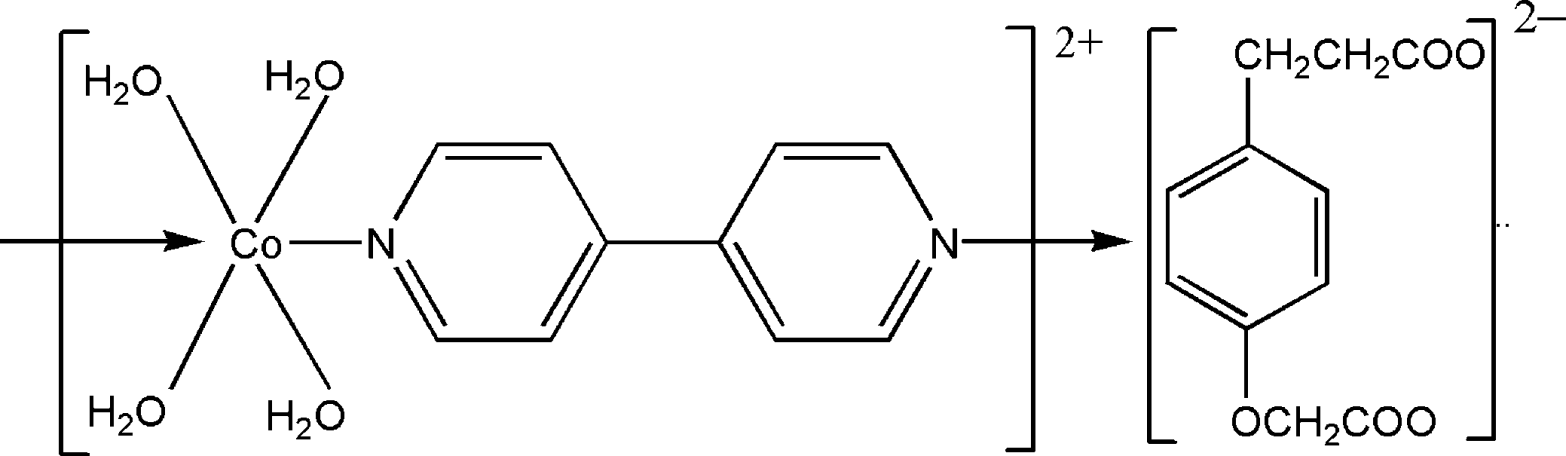


## Experimental

### 

#### Crystal data

[Co(C_10_H_8_N_2_)(H_2_O)_4_](C_11_H_10_O_5_)
*M* *_r_* = 509.37
Triclinic, 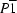

*a* = 7.1311 (10) Å
*b* = 7.6319 (10) Å
*c* = 10.4978 (14) Å
α = 91.930 (1)°
β = 101.832 (1)°
γ = 94.002 (1)°
*V* = 557.15 (13) Å^3^
*Z* = 1
Mo *K*α radiation
μ = 0.83 mm^−1^
*T* = 291 K
0.50 × 0.41 × 0.21 mm

#### Data collection

Bruker SMART CCD diffractometer
Absorption correction: multi-scan (*SADABS*; Sheldrick, 1996 ▶) *T*_min_ = 0.674, *T*_max_ = 0.845
4108 measured reflections
2036 independent reflections
2008 reflections with *I* > 2σ(*I*)
*R*_int_ = 0.011

#### Refinement

*R*[*F* ^2^ > 2σ(*F* ^2^)] = 0.036
*wR*(*F* ^2^) = 0.085
*S* = 1.06
2036 reflections
176 parameters
364 restraints
H-atom parameters constrained
Δρ_max_ = 0.61 e Å^−3^
Δρ_min_ = −0.63 e Å^−3^

### 

Data collection: *SMART* (Bruker, 1998 ▶); cell refinement: *SAINT* (Bruker, 1998 ▶); data reduction: *SAINT*; program(s) used to solve structure: *SHELXS97* (Sheldrick, 2008 ▶); program(s) used to refine structure: *SHELXL97* (Sheldrick, 2008 ▶); molecular graphics: *SHELXTL* (Sheldrick, 2008 ▶); software used to prepare material for publication: *SHELXTL*.

## Supplementary Material

Crystal structure: contains datablocks global, I. DOI: 10.1107/S1600536809021552/lh2836sup1.cif

Structure factors: contains datablocks I. DOI: 10.1107/S1600536809021552/lh2836Isup2.hkl

Additional supplementary materials:  crystallographic information; 3D view; checkCIF report

## Figures and Tables

**Table:** 

Co1—O1	2.0840 (16)
Co1—O2	2.1083 (16)
Co1—N1	2.1530 (17)

**Table:** 

O1^i^—Co1—O1	180
O1—Co1—O2^i^	88.34 (7)
O1—Co1—O2	91.66 (7)
O2^i^—Co1—O2	180
O1—Co1—N1	91.93 (7)
O2—Co1—N1	90.36 (7)
O1—Co1—N1^i^	88.07 (7)
O2—Co1—N1^i^	89.63 (7)
N1—Co1—N1^i^	180

Symmetry code: (i) 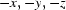.

**Table 2:** Hydrogen-bond geometry (Å, °)

*D*—H⋯*A*	*D*—H	H⋯*A*	*D*⋯*A*	*D*—H⋯*A*
O2—H4*W*⋯O4′^ii^	0.83	2.00	2.796 (10)	161
O2—H4*W*⋯O4^ii^	0.83	1.86	2.667 (10)	165
O2—H3*W*⋯O4^iii^	0.83	1.96	2.765 (14)	163
O2—H3*W*⋯O4′^iii^	0.83	1.86	2.678 (14)	170
O1—H2*W*⋯O3^iv^	0.82	2.07	2.868 (16)	164
O1—H2*W*⋯O3′^iv^	0.82	1.90	2.691 (16)	161
O1—H1*W*⋯O3^v^	0.83	1.97	2.789 (13)	169
O1—H1*W*⋯O3′^v^	0.83	1.79	2.612 (14)	174

Symmetry codes: (ii) 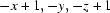; (iii) 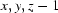; (iv) 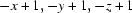; (v) 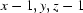.

**Fig. 1.**
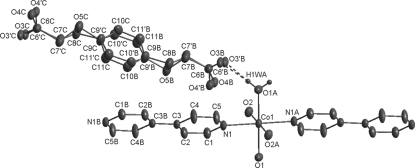
: Part of the title complex (I), showing the atom-labelling scheme. Displacement ellipsoids are drawn at the 30% probability level and most H atoms are omitted for clarity. Primed atoms indicate one of the disorder components. [Symmetry codes: (A) -x, -y, -z; (B) 1 - x, -y, 1 - z; (C) 1 + x, -1 + y, z.]

## supplementary crystallographic information

### Comment

In recent years, assembly of high-dimensional supramolecular coordination polymers *via* coordination bonds, hydrogen bonds, and π···π stacks (Ye *et al.*, 2005) have received much attention and carboxylic acid compounds as good donors and acceptors of hydrogen bonds have been widely utilized as ligands. 3-(4-hydroxyphenyl)propanoic acid (Tan *et al.*, 2007) a pseudo-symmetric carboxylate acid is a potential multidentate ligand and a good donor and acceptor of hydrogen bonds, but its coordination polymers are less investigated. 4,4'-bipyridine is a neutral linear bifunctional ligand that is widely used as an excellent spacer in the construction of supramolecular architectures (Cussen *et al.*, 2002; Tao *et al.*, 2000). Here, we report the synthesis and crystals structure of a cobalt supramolecular complex formed using with 4,4'-bipyridine and 3-(4-(carboxymethoxy)phenyl)propanoic acid.

The asymmetric unit and some symmetry related atoms  are shown in Fig. 1. The unique Co^II^ ion lies on an invesion center and is coordinated by two nitrogen atoms from two 4,4'-bipyridine ligands and four oxygen atoms from four water molecules in a slightly distorted octahedral coordination geometry. The molecules of 3-(4-(carboxymethoxy)phenyl)propanoic acid are completely deprotonated but remain uncoordinated, and the 4,4'-bipyridine ligands act as bridging to join the Co^II^ ions into a one-dimensional chain structure, which is further linked to a 3-D network through intermolecular O—H···O hydrogen bonds.

### Experimental

A mixture of CoCl_2_.2H_2_O (0.1 mmol), 4,4'-bipyridine (0.1 mmol), 3-(4-(carboxymethoxy)phenyl)propanoic acid (0.1 mmol) and 10 ml water was placed in a tube and heated at 363 K for 6 h, then cooled to room temperature. Upon cooling to RT, a few red crystals were obtained. Anal. Calcd for C21H26N2O9Co (509.37): C, 49.47; H, 5.10; N, 5.50%; Found: C, 49.23; H, 4.98; N, 5.19%.

### Refinement

The water H atoms were located in a difference Fourier map but were included in fixed positions in riding-model approximation with the O—H distances in the range 0.8245–0.8271 Å and *U*_iso_(H) = 1.5U_eq_(O); all other H atoms were placed in geometrically idealized positions with *C*—*H*(methylene) = 0.97 Å, C—H(aromatic) = 0.93 Å, and *U*_iso_(H) = 1.2U_eq_(C). The dianion is completely disordered over an inversion center. The SADI and EADP commands in SHELXL (Sheldrick, 2008) were used to model the disorder.

### Crystal data

**Table:**

[Co(C_10_H_8_N_2_)(H_2_O)_4_](C_11_H_10_O_5_)	*Z* = 1
*M**_r_* = 509.37	*F*(000) = 265
Triclinic, *P*1	*D*_x_ = 1.518 Mg m^−^^3^
Hall symbol: -P 1	Mo *K*α radiation, λ = 0.71073 Å
*a* = 7.1311 (10) Å	Cell parameters from 3868 reflections
*b* = 7.6319 (10) Å	θ = 2.7–28.2°
*c* = 10.4978 (14) Å	µ = 0.83 mm^−^^1^
α = 91.930 (1)°	*T* = 291 K
β = 101.832 (1)°	Block, red
γ = 94.002 (1)°	0.50 × 0.41 × 0.21 mm
*V* = 557.15 (13) Å^3^	

### Data collection

**Table:**

Bruker SMART CCD diffractometer	2036 independent reflections
Radiation source: fine-focus sealed tube	2008 reflections with *I* > 2σ(*I*)
graphite	*R*_int_ = 0.011
φ and ω scans	θ_max_ = 25.5°, θ_min_ = 2.7°
Absorption correction: multi-scan (*SADABS*; Sheldrick, 1996)	*h* = −8→8
*T*_min_ = 0.674, *T*_max_ = 0.845	*k* = −9→9
4108 measured reflections	*l* = −12→12

### Refinement

**Table:**

Refinement on *F*^2^	Primary atom site location: structure-invariant direct methods
Least-squares matrix: full	Secondary atom site location: difference Fourier map
*R*[*F*^2^ > 2σ(*F*^2^)] = 0.036	Hydrogen site location: inferred from neighbouring sites
*wR*(*F*^2^) = 0.085	H-atom parameters constrained
*S* = 1.06	*w* = 1/[σ^2^(*F*_o_^2^) + (0.0304*P*)^2^ + 0.7695*P*] where *P* = (*F*_o_^2^ + 2*F*_c_^2^)/3
2036 reflections	(Δ/σ)_max_ < 0.001
176 parameters	Δρ_max_ = 0.61 e Å^−^^3^
364 restraints	Δρ_min_ = −0.63 e Å^−^^3^

### Special details

**Table:**

Geometry. All e.s.d.'s (except the e.s.d. in the dihedral angle between two l.s. planes) are estimated using the full covariance matrix. The cell e.s.d.'s are taken into account individually in the estimation of e.s.d.'s in distances, angles and torsion angles; correlations between e.s.d.'s in cell parameters are only used when they are defined by crystal symmetry. An approximate (isotropic) treatment of cell e.s.d.'s is used for estimating e.s.d.'s involving l.s. planes.
Refinement. Refinement of *F*^2^ against ALL reflections. The weighted *R*-factor *wR* and goodness of fit *S* are based on *F*^2^, conventional *R*-factors *R* are based on *F*, with *F* set to zero for negative *F*^2^. The threshold expression of *F*^2^ > σ(*F*^2^) is used only for calculating *R*-factors(gt) *etc*. and is not relevant to the choice of reflections for refinement. *R*-factors based on *F*^2^ are statistically about twice as large as those based on *F*, and *R*- factors based on ALL data will be even larger.

### Fractional atomic coordinates and isotropic or equivalent isotropic displacement parameters (Å^2^)

**Table:**

	*x*	*y*	*z*	*U*_iso_*/*U*_eq_	Occ. (<1)
O3	0.7094 (13)	0.448 (3)	0.8766 (14)	0.0419 (15)	0.50
O4	0.522 (2)	0.2444 (14)	0.9494 (9)	0.0368 (16)	0.50
C8	0.3511 (11)	0.4124 (10)	0.6667 (8)	0.0635 (11)	0.50
H8A	0.4539	0.4806	0.6385	0.076*	0.50
H8B	0.3700	0.2890	0.6533	0.076*	0.50
C6	0.5492 (16)	0.370 (3)	0.879 (3)	0.0363 (9)	0.50
C7	0.3670 (11)	0.4520 (12)	0.8095 (7)	0.0522 (14)	0.50
H7A	0.2547	0.4010	0.8376	0.063*	0.50
H7B	0.3781	0.5780	0.8285	0.063*	0.50
C9	0.1644 (19)	0.451 (2)	0.5829 (11)	0.0583 (13)	0.50
C10	0.1182 (18)	0.3869 (16)	0.4541 (10)	0.0612 (15)	0.50
H10	0.1933	0.3074	0.4232	0.073*	0.50
C11	0.0411 (14)	0.5566 (15)	0.6274 (14)	0.0575 (17)	0.50
H11	0.0658	0.5913	0.7154	0.069*	0.50
O3'	0.7312 (13)	0.450 (3)	0.9043 (14)	0.0419 (15)	0.50
O4'	0.513 (2)	0.2290 (14)	0.9156 (9)	0.0368 (16)	0.50
C6'	0.5648 (17)	0.374 (3)	0.876 (3)	0.0363 (9)	0.50
C7'	0.4168 (11)	0.4553 (12)	0.7713 (8)	0.0522 (14)	0.50
H7'1	0.3503	0.5373	0.8153	0.063*	0.50
H7'2	0.4868	0.5234	0.7173	0.063*	0.50
O5	0.2825 (7)	0.3461 (6)	0.6915 (5)	0.0635 (11)	0.50
C9'	0.1435 (18)	0.435 (2)	0.5964 (11)	0.0583 (13)	0.50
C10'	0.1492 (18)	0.4241 (16)	0.4648 (9)	0.0612 (15)	0.50
H10'	0.2554	0.3795	0.4403	0.073*	0.50
C11'	−0.0013 (14)	0.5223 (14)	0.6328 (14)	0.0575 (17)	0.50
H11'	−0.0012	0.5453	0.7204	0.069*	0.50
Co1	0.0000	0.0000	0.0000	0.02210 (13)	
O1	0.0283 (2)	0.2722 (2)	−0.01206 (18)	0.0396 (4)	
H1W	−0.0701	0.3243	−0.0349	0.059*	
H2W	0.1207	0.3397	0.0241	0.059*	
O2	0.2400 (2)	−0.0305 (2)	−0.08486 (16)	0.0338 (4)	
H3W	0.3136	0.0561	−0.0905	0.051*	
H4W	0.2965	−0.1087	−0.0441	0.051*	
N1	0.1824 (3)	0.0139 (3)	0.19142 (17)	0.0288 (4)	
C1	0.3560 (3)	0.1002 (3)	0.2175 (2)	0.0341 (5)	
H1	0.3934	0.1655	0.1524	0.041*	
C2	0.4835 (3)	0.0981 (3)	0.3358 (2)	0.0356 (6)	
H2	0.6033	0.1601	0.3483	0.043*	
C3	0.4332 (3)	0.0036 (3)	0.4357 (2)	0.0285 (5)	
C4	0.2514 (4)	−0.0850 (5)	0.4090 (3)	0.0557 (9)	
H4	0.2096	−0.1502	0.4726	0.067*	
C5	0.1325 (4)	−0.0761 (4)	0.2877 (3)	0.0532 (8)	
H5	0.0114	−0.1361	0.2725	0.064*	

### Atomic displacement parameters (Å^2^)

**Table:**

	*U*^11^	*U*^22^	*U*^33^	*U*^12^	*U*^13^	*U*^23^
O3	0.0288 (17)	0.0355 (10)	0.050 (5)	−0.0043 (18)	−0.0169 (18)	0.009 (3)
O4	0.0306 (14)	0.0374 (17)	0.038 (4)	0.0015 (14)	−0.005 (3)	0.010 (3)
C8	0.053 (2)	0.056 (2)	0.067 (2)	0.0142 (17)	−0.0250 (18)	0.0040 (18)
C6	0.0301 (17)	0.0288 (13)	0.0428 (15)	0.0016 (14)	−0.0095 (18)	0.0037 (10)
C7	0.044 (3)	0.0434 (16)	0.059 (3)	0.006 (2)	−0.016 (2)	0.011 (2)
C9	0.044 (2)	0.058 (2)	0.065 (2)	0.0198 (19)	−0.0110 (16)	−0.0018 (17)
C10	0.042 (3)	0.063 (3)	0.072 (2)	0.020 (3)	−0.0052 (19)	−0.016 (2)
C11	0.047 (3)	0.063 (3)	0.059 (2)	0.010 (3)	0.001 (2)	−0.001 (2)
O3'	0.0288 (17)	0.0355 (10)	0.050 (5)	−0.0043 (18)	−0.0169 (18)	0.009 (3)
O4'	0.0306 (14)	0.0374 (17)	0.038 (4)	0.0015 (14)	−0.005 (3)	0.010 (3)
C6'	0.0301 (17)	0.0288 (13)	0.0428 (15)	0.0016 (14)	−0.0095 (18)	0.0037 (10)
C7'	0.044 (3)	0.0434 (16)	0.059 (3)	0.006 (2)	−0.016 (2)	0.011 (2)
O5	0.053 (2)	0.056 (2)	0.067 (2)	0.0142 (17)	−0.0250 (18)	0.0040 (18)
C9'	0.044 (2)	0.058 (2)	0.065 (2)	0.0198 (19)	−0.0110 (16)	−0.0018 (17)
C10'	0.042 (3)	0.063 (3)	0.072 (2)	0.020 (3)	−0.0052 (19)	−0.016 (2)
C11'	0.047 (3)	0.063 (3)	0.059 (2)	0.010 (3)	0.001 (2)	−0.001 (2)
Co1	0.0185 (2)	0.0246 (2)	0.0200 (2)	0.00069 (15)	−0.00368 (15)	0.00413 (15)
O1	0.0309 (9)	0.0260 (8)	0.0538 (11)	−0.0002 (7)	−0.0092 (8)	0.0043 (8)
O2	0.0249 (8)	0.0398 (9)	0.0356 (9)	0.0033 (7)	0.0029 (7)	0.0085 (7)
N1	0.0246 (9)	0.0354 (10)	0.0226 (9)	0.0004 (8)	−0.0041 (7)	0.0048 (8)
C1	0.0307 (12)	0.0427 (14)	0.0248 (11)	−0.0057 (10)	−0.0024 (9)	0.0090 (10)
C2	0.0279 (12)	0.0450 (14)	0.0281 (12)	−0.0084 (10)	−0.0052 (9)	0.0073 (10)
C3	0.0280 (11)	0.0307 (11)	0.0229 (11)	0.0029 (9)	−0.0044 (9)	0.0025 (9)
C4	0.0417 (15)	0.084 (2)	0.0310 (14)	−0.0249 (15)	−0.0109 (11)	0.0275 (14)
C5	0.0352 (14)	0.079 (2)	0.0348 (14)	−0.0234 (14)	−0.0105 (11)	0.0218 (14)

### Geometric parameters (Å, °)

**Table:**

O3—C6	1.257 (6)	C10'—H10'	0.9300
O4—C6	1.257 (6)	C11'—C10'^i^	1.41 (2)
C8—C9	1.491 (13)	C11'—H11'	0.9300
C8—C7	1.499 (11)	Co1—O1^ii^	2.0840 (16)
C8—H8A	0.9700	Co1—O1	2.0840 (16)
C8—H8B	0.9700	Co1—O2^ii^	2.1083 (16)
C6—C7	1.540 (8)	Co1—O2	2.1083 (16)
C7—H7A	0.9700	Co1—N1	2.1530 (17)
C7—H7B	0.9700	Co1—N1^ii^	2.1531 (17)
C9—C11	1.375 (6)	O1—H1W	0.8271
C9—C10	1.387 (7)	O1—H2W	0.8245
C10—C11^i^	1.379 (19)	O2—H3W	0.8262
C10—H10	0.9300	O2—H4W	0.8251
C11—C10^i^	1.379 (19)	N1—C1	1.333 (3)
C11—H11	0.9300	N1—C5	1.334 (3)
O3'—C6'	1.258 (6)	C1—C2	1.382 (3)
O4'—C6'	1.255 (6)	C1—H1	0.9300
C6'—C7'	1.537 (7)	C2—C3	1.385 (3)
C7'—O5	1.352 (10)	C2—H2	0.9300
C7'—H7'1	0.9700	C3—C4	1.390 (3)
C7'—H7'2	0.9700	C3—C3^iii^	1.489 (4)
O5—C9'	1.475 (13)	C4—C5	1.384 (3)
C9'—C11'	1.377 (6)	C4—H4	0.9300
C9'—C10'	1.391 (7)	C5—H5	0.9300
C10'—C11'^i^	1.41 (2)		
			
C9—C8—C7	114.6 (7)	C9'—C11'—H11'	120.6
C9—C8—H8A	108.6	C10'^i^—C11'—H11'	120.6
C7—C8—H8A	108.6	O1^ii^—Co1—O1	180
C9—C8—H8B	108.6	O1^ii^—Co1—O2^ii^	91.66 (7)
C7—C8—H8B	108.6	O1—Co1—O2^ii^	88.34 (7)
H8A—C8—H8B	107.6	O1^ii^—Co1—O2	88.33 (7)
O3—C6—O4	125.4 (9)	O1—Co1—O2	91.66 (7)
O3—C6—C7	118.0 (7)	O2^ii^—Co1—O2	180
O4—C6—C7	115.7 (8)	O1^ii^—Co1—N1	88.07 (7)
C8—C7—C6	106.0 (13)	O1—Co1—N1	91.93 (7)
C8—C7—H7A	110.5	O2^ii^—Co1—N1	89.64 (7)
C6—C7—H7A	110.5	O2—Co1—N1	90.36 (7)
C8—C7—H7B	110.5	O1^ii^—Co1—N1^ii^	91.93 (7)
C6—C7—H7B	110.5	O1—Co1—N1^ii^	88.07 (7)
H7A—C7—H7B	108.7	O2^ii^—Co1—N1^ii^	90.36 (7)
C11—C9—C10	118.6 (7)	O2—Co1—N1^ii^	89.63 (7)
C11—C9—C8	121.7 (6)	N1—Co1—N1^ii^	180
C10—C9—C8	119.5 (5)	Co1—O1—H1W	118.4
C11^i^—C10—C9	119.0 (12)	Co1—O1—H2W	126.8
C11^i^—C10—H10	120.5	H1W—O1—H2W	112.0
C9—C10—H10	120.5	Co1—O2—H3W	119.7
C9—C11—C10^i^	122.2 (12)	Co1—O2—H4W	104.4
C9—C11—H11	118.9	H3W—O2—H4W	112.1
C10^i^—C11—H11	118.9	C1—N1—C5	116.50 (19)
O4'—C6'—O3'	125.8 (9)	C1—N1—Co1	122.27 (15)
O4'—C6'—C7'	116.6 (7)	C5—N1—Co1	121.02 (16)
O3'—C6'—C7'	117.2 (6)	N1—C1—C2	123.7 (2)
O5—C7'—C6'	118.2 (10)	N1—C1—H1	118.2
O5—C7'—H7'1	107.8	C2—C1—H1	118.2
C6'—C7'—H7'1	107.8	C1—C2—C3	120.1 (2)
O5—C7'—H7'2	107.8	C1—C2—H2	119.9
C6'—C7'—H7'2	107.8	C3—C2—H2	119.9
H7'1—C7'—H7'2	107.1	C2—C3—C4	116.2 (2)
C7'—O5—C9'	114.6 (8)	C2—C3—C3^iii^	121.9 (3)
C11'—C9'—C10'	117.9 (7)	C4—C3—C3^iii^	121.9 (3)
C11'—C9'—O5	121.8 (6)	C5—C4—C3	120.0 (2)
C10'—C9'—O5	120.2 (6)	C5—C4—H4	120.0
C9'—C10'—C11'^i^	122.8 (12)	C3—C4—H4	120.0
C9'—C10'—H10'	118.6	N1—C5—C4	123.5 (2)
C11'^i^—C10'—H10'	118.6	N1—C5—H5	118.2
C9'—C11'—C10'^i^	118.8 (12)	C4—C5—H5	118.2
			
C9—C8—C7—C6	168.9 (10)	O1—Co1—N1—C1	−51.9 (2)
O3—C6—C7—C8	76 (3)	O2^ii^—Co1—N1—C1	−140.20 (19)
O4—C6—C7—C8	−114 (2)	O2—Co1—N1—C1	39.80 (19)
C7—C8—C9—C11	17.1 (18)	N1^ii^—Co1—N1—C1	66 (3)
C7—C8—C9—C10	−167.0 (13)	O1^ii^—Co1—N1—C5	−46.4 (2)
C11—C9—C10—C11^i^	5(2)	O1—Co1—N1—C5	133.6 (2)
C8—C9—C10—C11^i^	−170.8 (13)	O2^ii^—Co1—N1—C5	45.3 (2)
C10—C9—C11—C10^i^	−5(2)	O2—Co1—N1—C5	−134.7 (2)
C8—C9—C11—C10^i^	170.5 (13)	N1^ii^—Co1—N1—C5	−109 (3)
O4'—C6'—C7'—O5	−27 (3)	C5—N1—C1—C2	1.0 (4)
O3'—C6'—C7'—O5	146 (2)	Co1—N1—C1—C2	−173.7 (2)
C6'—C7'—O5—C9'	178.6 (13)	N1—C1—C2—C3	−0.5 (4)
C7'—O5—C9'—C11'	−74.6 (15)	C1—C2—C3—C4	−0.2 (4)
C7'—O5—C9'—C10'	109.2 (15)	C1—C2—C3—C3^iii^	179.5 (3)
C11'—C9'—C10'—C11'^i^	−8(2)	C2—C3—C4—C5	0.3 (5)
O5—C9'—C10'—C11'^i^	168.0 (12)	C3^iii^—C3—C4—C5	−179.4 (3)
C10'—C9'—C11'—C10'^i^	8(2)	C1—N1—C5—C4	−0.9 (5)
O5—C9'—C11'—C10'^i^	−168.3 (12)	Co1—N1—C5—C4	173.9 (3)
O1^ii^—Co1—N1—C1	128.1 (2)	C3—C4—C5—N1	0.3 (6)

Symmetry codes: (i) −*x*, −*y*+1, −*z*+1; (ii) −*x*, −*y*, −*z*; (iii) −*x*+1, −*y*, −*z*+1.

### Hydrogen-bond geometry (Å, °)

**Table:**

*D*—H···*A*	*D*—H	H···*A*	*D*···*A*	*D*—H···*A*
O2—H4W···O4'^iii^	0.83	2.00	2.796 (10)	161
O2—H4W···O4^iii^	0.83	1.86	2.667 (10)	165
O2—H3W···O4^iv^	0.83	1.96	2.765 (14)	163
O2—H3W···O4'^iv^	0.83	1.86	2.678 (14)	170
O1—H2W···O3^v^	0.82	2.07	2.868 (16)	164
O1—H2W···O3'^v^	0.82	1.90	2.691 (16)	161
O1—H1W···O3^vi^	0.83	1.97	2.789 (13)	169
O1—H1W···O3'^vi^	0.83	1.79	2.612 (14)	174

Symmetry codes: (iii) −*x*+1, −*y*, −*z*+1; (iv) *x*, *y*, *z*−1; (v) −*x*+1, −*y*+1, −*z*+1; (vi) *x*−1, *y*, *z*−1.
